# Leukocyte-Reduced Platelet-Rich Plasma Treatment of Basal Thumb Arthritis: A Pilot Study

**DOI:** 10.1155/2016/9262909

**Published:** 2016-07-05

**Authors:** Markus Loibl, Siegmund Lang, Lena-Marie Dendl, Michael Nerlich, Peter Angele, Sebastian Gehmert, Michaela Huber

**Affiliations:** ^1^Department of Trauma Surgery, University Medical Center Regensburg, 93053 Regensburg, Germany; ^2^Institute of Radiology, University Medical Center Regensburg, 93053 Regensburg, Germany; ^3^Department of Orthopedic Surgery, University Hospital Basel, 4056 Basel, Switzerland

## Abstract

A positive effect of intra-articular platelet-rich plasma (PRP) injection has been discussed for osteoarthritic joint conditions in the last years. The purpose of this study was to evaluate PRP injection into the trapeziometacarpal (TMC) joint. We report about ten patients with TMC joint osteoarthritis (OA) that were treated with 2 intra-articular PRP injections 4 weeks apart. PRP was produced using the Double Syringe System (Arthrex Inc., Naples, Florida, USA). A total volume of 1.47 ± 0.25 mL PRP was injected at the first injection and 1.5 ± 0.41 mL at the second injection, depending on the volume capacity of the joint. Patients were evaluated using VAS, strength measures, and the Mayo Wrist score and DASH score after 3 and 6 months. VAS significantly decreased from 6.2 ± 1.6 to 5.4 ± 2.2 at six-month follow-up (*P* < 0.05). The DASH score was unaffected; however, the Mayo Wrist score significantly improved from 46.5 ± 18.6 to 67.5 ± 19.0 at six-month follow-up (*P* = 0.05). Grip was unaffected, whereas pinch declined from 6.02 ± 2.99 to 3.96 ± 1.77 at six-month follow-up (*P* < 0.05). We did not observe adverse events after the injection of PRP, except one occurrence of a palmar wrist ganglion, which resolved without treatment. PRP injection for symptomatic TMC OA is a reasonable therapeutic option in early stages TMC OA and can be performed with little to no morbidity.

## 1. Introduction

Several conservative and operative techniques have been described for the treatment of trapeziometacarpal (TMC) joint osteoarthritis (OA) over the last 70 years. In general, OA involves a perturbed joint environment at the cellular level with alterations in the composition of the synovial fluid. As a consequence, chondrocytes become “activated” with increased proliferation, production of matrix-degrading enzymes, cytokines, and cytokine receptors [1]. Finally, the inadequate healing response to synovial inflammation results in further structural cartilage degradation [2].

Besides splint and exercise regimes, hyaluronate injections have been evaluated as conservative treatment in placebo-controlled randomized trials for symptomatic treatment of basal thumb arthritis [3, 4]. All performed operative procedures, like trapeziectomy, trapeziectomy with ligament reconstruction, arthrodesis, or implant arthroplasty, demonstrated good clinical results [5, 6].

Platelet-rich plasma (PRP) is an autologous blood product that contains an increased concentration of platelets and emerged as a safe treatment modality to accelerate healing of musculoskeletal injuries [7]. Platelets contain more than 5000 proteins, of which more than 300 are released upon activation [8]. Particularly, among these bioactive proteins are growth factors and cytokines.

PRP injection into joints can modify the biological microenvironment inside the joint. Thereby, PRP affects local and infiltrating cells, mainly synovial cells, endothelial cells, immune cells, and cellular components of cartilage and bone [9]. Ultimately, it is believed to reduce the inflammatory process and alter the joint homeostasis of anabolism and catabolism in cartilage [10]. Numerous PRP formulations are used in experimental and clinical research and yield products with different cellular compositions and biological characteristics [11]. Leukocyte-reduced PRP has proven superior over leukocyte-rich PRP in the treatment of OA in vitro. It has been shown that the interaction of white blood cells with chondrocytes and synoviocytes results in a significantly higher the release of proinflammatory cytokines IL-1b and IL-6 [12, 13]. For this reason, we recently characterized and optimized a leukocyte-reduced PRP for the intra-articular application [14]. We disabled the brake after the centrifugation process and achieved a further significant reduction of white blood cell content in PRP in comparison to PRP produced according to the manufacturer' instructions.

Three randomized hyaluronan-controlled trials [15–17] and one placebo-controlled clinical trial for OA of the knee joint [18] demonstrated decreased pain and improved function after PRP injections in patients with symptomatic knee OA. The implications for PRP treatment of OA in other joints are unknown. To our knowledge, there is no study in the literature investigating multiple PRP injections for the treatment of TMC joint OA.

The primary aim of this study was to gather first results on the clinical effects of PRP injections for the treatment of different stages of TMC OA. It was hypothesized that intra-articular treatment with leukocyte-reduced PRP would lead to improvements in pain and function of the TMC joint during the 6-month follow-up.

## 2. Methods

**Figure 1:**
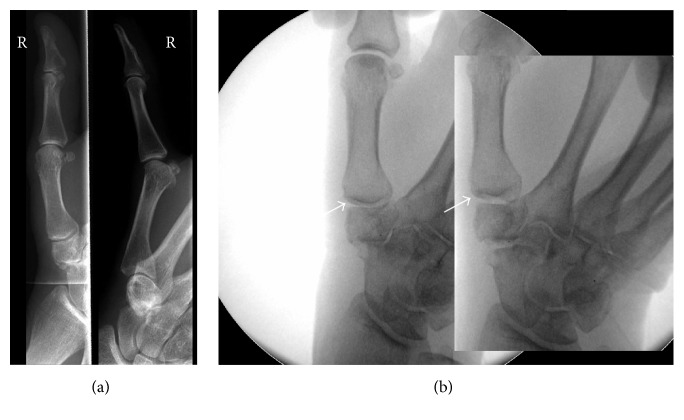
(a) Anteroposterior and lateral X-rays of the right TMC joint of a 71-year-old female with OA of the TMC and scaphotrapeziotrapezoid (STT) joints classified as Eaton and Littler III. The patient reported pain since 71 weeks and had undergone anti-inflammatory pain medication as needed since then. (b) Fluoroscopic images of the right hand before and after PRP injection resulting in TMC and STT joint distension due to intra-articular PRP application.

The study was approved by the local ethics committee of the University of Regensburg (15-104-0274). A total of ten patients with TMC OA were treated with 2 intra-articular PRP injections 4 weeks apart at the University Medical Center Regensburg (Figures 1(a) and 1(b)). Two patients had received a steroid or hyaluronan injection in the years before PRP treatment, both with short-term pain relief. The condition was diagnosed using standard radiographic and clinical criteria: basal joint tenderness, thumb or wrist pain at rest or with activity, joint stiffness, decreased mobility, deformity, instability, and decreased hand function. No splinting was used after each injection. Rest was recommended for 1-2 days with full range of motion as tolerated. During the course of treatment, all patients did not take corticosteroids or nonsteroidal anti-inflammatory drugs (NSAIDs).

### 2.1. Radiologic Classification

All X-rays have been evaluated and classified by a blinded radiologist with the Eaton and Littler Classification as follows [19]:

 I: normal joint appearance or less than one-third subluxation.
 II: decrease of joint space, osteophytes less than 2 mm, and one-third subluxation or more.
 III: advanced joint distraction, subchondral cysts and sclerosis, and osteophytes greater than 2 mm.
 IV: involvement of several joint surfaces.

### 2.2. PRP Samples

Venous blood (15 mL) was drawn directly into the Arthrex Double Syringe (Arthrex Inc., Naples, Florida, USA) for the production of autologous conditioned plasma (ACP) using a winged infusion set (Sarstedt AG & Co., Nümbrecht, Germany). The ACP double syringe was processed using a Hettich Rotofix 32a centrifuge at 1500 rpm for 4 minutes with brake disabled as characterized previously [14]. The whole blood was separated into two distinct layers by centrifugation, whereas a plasma layer appeared on the top and the red/white blood cell layer was apparent on the bottom. The plasma containing the platelets (PRP) was isolated by drawing the inner syringe according to the manufacturer' instructions. Our previous work revealed a concentration of platelets by approximately 2.4 times in PRP (567.6 ± 143.1  × 10^3^/*μ*L; 95% confidence interval: 514.2–621.1 × 10^3^/*μ*L) in comparison to venous blood (232.5 ± 45.7  × 10^3^/*μ*L; 95% confidence interval: 215.5–249.6 × 10^3^/*μ*L), whereas a significant reduction of white blood cells to a marginal concentration in PRP was observed [14].

Thereafter, PRP was injected under sterile conditions into the TMC joint under fluoroscopic guidance from the dorsal side by the senior author. No local anesthetic was used. All patients received an injection of 1-2 mL into the joint depending on the volume capacity of the joint. The injection was repeated after four weeks.

### 2.3. Outcome Measures and Follow-Up

Prior to the first injection, baseline outcome measures and descriptive statistics were collected prospectively for all patients. Descriptive statistics included age, gender, and hand dominance. Patients completed the validated DASH questionnaire and a visual analog scale (VAS) for pain with activity. Moreover, the Mayo Wrist score was included. The grip strength and pinch strength were measured 3 times and the mean value was calculated. All patients were scheduled for follow-up visits with identical evaluation at 3 and 6 months after the first injection.

Statistical analysis was performed using SPSS software package (version 20, IBM SPSS, Chicago, Illinois), whereas all graphs were prepared by using GraphPad Prism (version 5, Statcon, La Jolla, California). All data were tested for normal distribution applying the Shapiro-Wilk test. Paired *t*-test and One-Way Analysis of Variance (ANOVA) with Bonferroni correction were used to analyze all normally distributed parameters (DASH score, Mayo Wrist score, and pinch and grip power) depending on the time of examination (first examination and 3 months and 6 months after treatment). The Wilcoxon Signed-Rank test with Bonferroni correction was applied to analyze differences between the VAS outcomes. Differences between groups, based on the Eaton and Littler Classification, were investigated by the One-Way Analysis of Variance (ANOVA) and Wilcoxon Signed-Rank test with Bonferroni correction. The Spearman Correlation test was used to analyze correlations between all parameters. Descriptive data are expressed in terms of mean ± standard deviation. The level of significance was set at *P* = 0.05 for all statistical tests.

## 3. Results

Ten patients were identified for this study. The mean age of the included patients was 56.1 ± 9.9 years at the time of the first injection. All patients were followed up for 6 months. The study cohort comprised 8 women and 2 men with involvement of the dominant hand in 3 patients and the nondominant hand in 7 patients (Table 1). A total volume of 1.47 ± 0.25 mL PRP was injected into the TMC joint at the first injection and 1.5 ± 0.41 mL at the second injection.

### 3.1. Clinical Outcome

At the time of the first injection, patients reported pain with a VAS of 6.2 ± 1.6, which significantly decreased to 4.0 ± 2.4 at three-month follow-up and 5.4 ± 2.2 at six-month follow-up (both *P* < 0.05). VAS increased significantly by 1.4 points from three- to six-month follow-up (*P* < 0.05). The DASH score remained similar with 32.9 ± 11.9 at baseline and 20.4 ± 14.7 at three-month and 26.8 ± 18.9 at 6-month follow-up (*P* ≥ 0.24). The Mayo Wrist score significantly improved from 46.5 ± 18.6 to 68.3 ± 18.5 at three-month follow-up (*P* = 0.05) and to 67.5 ± 19.0 at six-month follow-up (*P* = 0.05) (Table 2). Overall, 2 patients were very satisfied with the result of the treatment, 5 were satisfied, 3 patients indicated neither satisfied nor unsatisfied, and no patient was dissatisfied.

### 3.2. Trapeziometacarpal Osteoarthritis-Depending Results

To analyze the influence of severity of TMC OA according to the Eaton and Littler Classification, we created 4 subgroups of patients classified as Eaton and Littler I to IV. Two patients were classified as Eaton II, three patients as Eaton III, and five patients as Eaton IV. We found a positive correlation between patients' age and severity of TMC OA according to the Eaton and Littler Classification (*r* = 0.6, *P* < 0.01).

**Figure 2:**
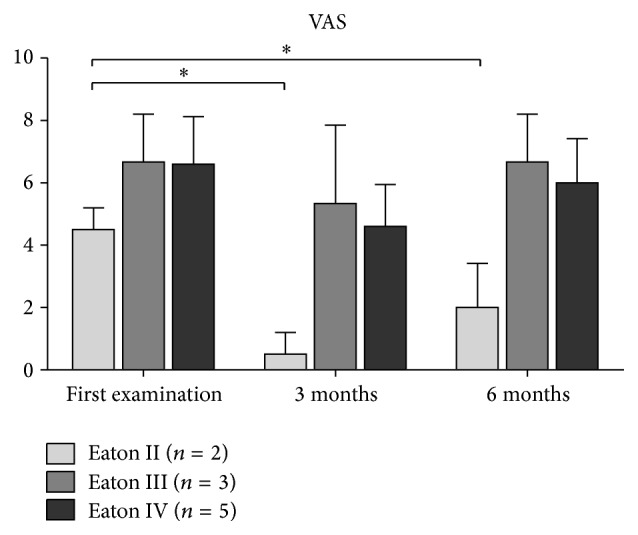
The course of visual analog scale (VAS) for pain depending on the severity of TMC OA. Data expressed as mean ± standard deviation. ^*∗*^ *P* ≤ 0.05.

**Figure 3 fig3:**
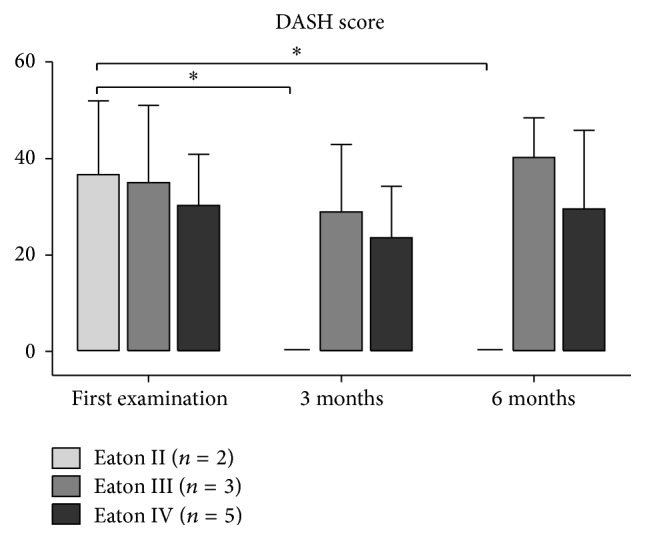
The course of the DASH score depending on the severity of TMC OA. Data expressed as mean ± standard deviation. ^*∗*^ *P* ≤ 0.05.

**Figure 4:**
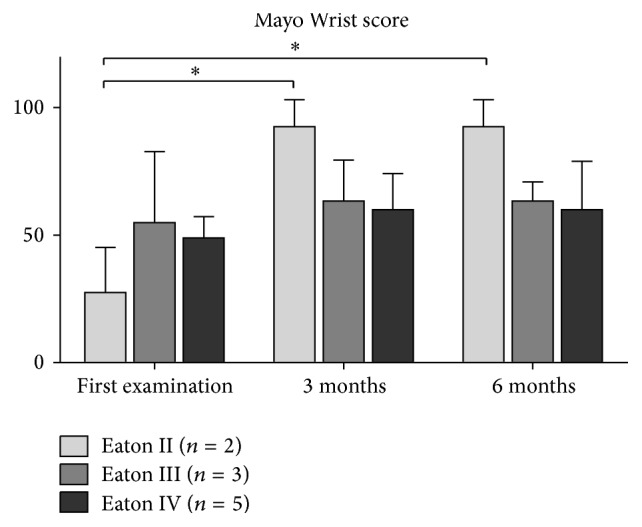
The course of the Mayo Wrist score depending on the severity of TMC OA. Data expressed as mean ± standard deviation. *∗* represents *P* < 0.05.

**Figure 5:**
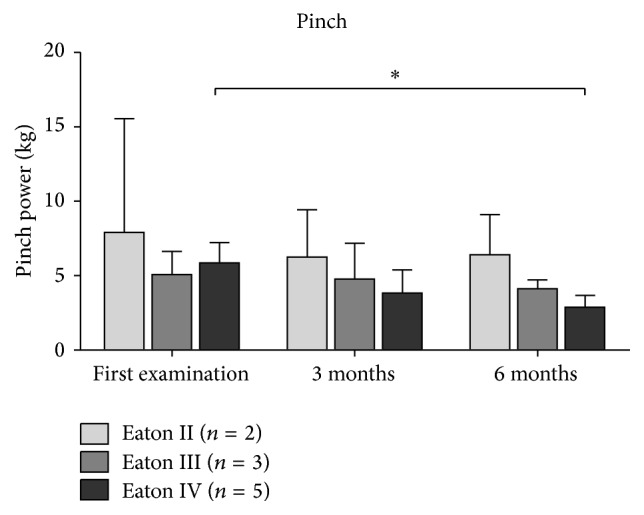
The course of the pinch depending on the severity of TMC OA. Data expressed as mean ± standard deviation. *∗* represents *P* < 0.05.

**Figure 6:**
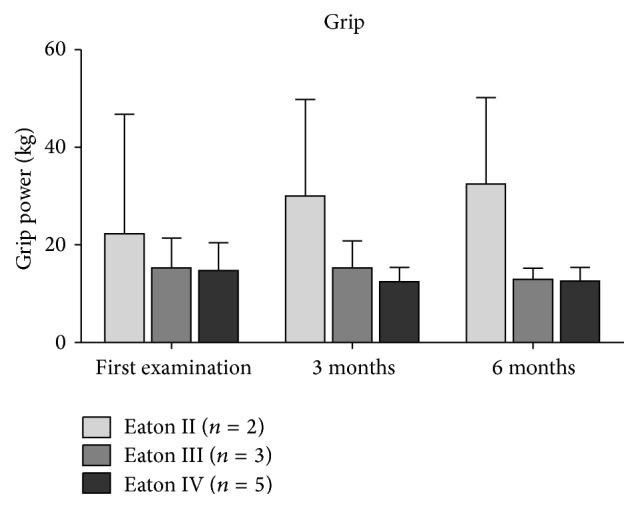
The course of the grip depending on the severity of TMC OA. Data expressed as mean ± standard deviation.

Patients with moderate OA graded as Eaton and Littler II reported pain with a VAS of 4.5 ± 0.7, which significantly decreased to 0.5 ± 0.7 at three-month and 2.0 ± 1.4 at six-month follow-up (both *P* < 0.05) (Figure 2). Moreover, the DASH score and Mayo Wrist score significantly improved, both at three- and six-month follow-up (all *P* ≤ 0.05) with 36.7 ± 15.3 to 0.0 ± 0.0 and 27.5 ± 17.7 to 92.5 ± 10.6, respectively (Figures 3 and 4). The strength measures pinch and grip were not affected by the PRP treatment at three and six months (all *P* = 1.0) (Figures 5 and 6).

In patients with a more severe OA graded as Eaton and Littler III and IV, the reported outcome measures VAS, the DASH score, and Mayo Wrist score did not change as a result of the PRP treatment (all *P* ≥ 0.06) (Figures 2 [Fig fig3]–4). Similarly, the strength measures did not improve (all *P* = 1.0). Looking at patients graded as Eaton IV, the pinch even decreased over time with 5.8 ± 1.4 kg at baseline, 3.8 ± 1.5 kg after three months (*P* = 0.089), and 2.9 ± 0.8 kg after six months (*P* = 0.01) (Figure 5).

## 4. Discussion

Several conservative treatment options have been evaluated in the treatment of TMC OA in the past [3]. PRP as an autologous blood-derived product can modify the biological microenvironment inside the joint by reducing the inflammatory process and recreate joint homeostasis within the inflamed joint [9]. Several clinical trials demonstrated decreased pain and improved function after PRP injections in patients with symptomatic OA of the knee joint [15–18]. Very little is known about the implications for PRP treatment of OA in other joints. Therefore, the primary aim of this study was to gather first results about the clinical effects of PRP injections for the treatment of different stages of TMC OA.

These first results indicate that intra-articular injections of PRP for TMC OA represent a safe conservative treatment modality. Patients with mild to moderate TMC OA experience persistent decreased pain at six-month follow-up after two intra-articular injections of PRP. Furthermore, these patients revealed a clinically significant improvement of the DASH score and Mayo Wrist score. Patients with a more severe OA graded as Eaton and Littler III and IV did not experience a persistent benefit.

In the current study, we did not observe adverse events after the injection of PRP, except one occurrence of a palmar wrist ganglion, which resolved without treatment. The injected PRP volume was depending on the size of the joint. PRP was injected until there was no possibility to add more into the joint. The applied intra-articular volume of about 1.5 mL was similar at both injections for every patient, however, less than in other studies investigating viscosupplementation [3, 20]. The resulting joint distension was supervised fluoroscopically and resolved after one month when the second injection was performed. Progressive instability of the TMC joint due to weakening of the articular capsule and ligaments can be observed after corticosteroid injections at rare intervals [20]. We did not observe progressive instability of the TMC joint in any of our patients. Considering the pathogenesis of primary TMC OA, further weakening of the capsular and ligament stabilizers should be prevented for successful treatment [21].

The most important finding of the present study was that patients demonstrated a significant pain relief after two PRP injections at 3- and 6-month follow-up. Patients with mild to moderate TMC OA, especially, experienced a persistent pain relief.

Patients classified as Eaton II had been free of pain after 6 months, and patients classified as Eaton III and IV had pain relief after 3 months, which did not fully retain up to 6 months. Similar studies about corticosteroid injection also report limited success for patients with late stages of TMC OA [22, 23].

We are aware of two prospective, randomized clinical trials that investigate the efficacy of viscosupplementation for TMC OA [3, 20]. Stahl et al. demonstrated significant improvement of strength tests after hyaluronan injection in comparison to corticosteroid injection at six-month follow-up. Similarly, Heyworth et al. demonstrated significant improvement of strength tests and pain when compared to baseline in the hyaluronan group only. In the current study, the strength measures did not improve. Looking at patients graded as Eaton IV, the pinch even decreased over time.

A few prospective studies on the effectiveness of PRP on knee degeneration revealed significant improvements in pain and clinical outcome [24]. Moreover, patients with early OA of the knee joint demonstrated significantly better clinical results with multiple PRP injections. Accordingly, patients with mild TMC OA experienced significant improvements of Mayo Wrist score and pain at 6-month follow-up. The better clinical results observed in patients with early OA could be explained by a better responsiveness to growth factors in less degenerated joints with more vital cells. Therefore, it was hypothesized that multiple PRP injections would yield an effective treatment option for early OA [15]. However, further studies with higher patient numbers will have to reproduce our first results and will have to elucidate on the number and frequency of PRP injections for effective treatment.

The presented study has a number of limitations: first of all, the study design lacks a control or placebo group. Second, the results of this clinical trial are based on the limited number of ten patients and therefore have to be interpreted with caution. Third, the subgroup analysis according on to the Eaton and Littler Classification based on radiographic features does not necessarily reflect the symptoms of pain experienced by the patient. However, the strengths of the present study are that all data were collected prospectively and comprise a detailed inquiry about clinical and functional outcome after intra-articular PRP injections for TMC OA.

## 5. Conclusion

At present, PRP injection into symptomatic OA of the TMC joint is a reasonable therapeutic option in early stages of TMC OA and can be performed with little to no morbidity. This study represents preliminary data to support PRP as another option in the conservative management of TMC OA to restore joint homeostasis in the inflamed joint. Further research should be conducted to confirm our findings and should address the value of autologous PRP injection versus viscosupplementation or steroid injection.

## Figures and Tables

**Table 1:** Baseline patient characteristics.

Age	56.1 ± 9.9 (49.3–62.7)
Female/male	8/2
Dominant hand	3
Eaton score II/III/IV	2/3/5
VAS	6.2 ± 1.6 (5.1–7.3)
DASH score	32.9 ± 11.9 (24.4–41.5)
Mayo Wrist score	46.5 ± 18.6 (33.2–59.8)
Pinch	6.0 ± 3.0 (3.9–8.2)
Grip	16.4 ± 9.9 (9.3–23.5)

Data expressed as mean ± standard deviation (95% confidence interval). VAS = visual analog scale.

**Table 2:** Clinical outcome.

	First examination	3 months	*P* value	6 months	*P* value
VAS	6.2 ± 1.6 (5.1–7.3)	4.0 ± 2.4 (2.3–5.7)	<0.05	5.4 ± 2.2 (3.8–7.0)	<0.05
DASH score	32.9 ± 11.9 (24.4–41.5)	20.4 ± 14.7 (10.0–30.1)	0.24	26.8 ± 18.9 (13.3–40.3)	1
Mayo Wrist score	46.5 ± 18.6 (33.2–59.8)	68.3 ± 18.5 (54.1–82.6)	0.05	67.5 ± 19.0 (53.9–81.1)	0.05
Pinch	6.0 ± 3.0 (3.9–8.2)	4.6 ± 2.1 (3.1–6.1)	0.12	4.9 ± 1.8 (2.7–5.2)	<0.05
Grip	16.4 ± 9.9 (9.3–23.5)	16.8 ± 10.2 (9.5–24.1)	0.83	16.7 ± 10.4 (9.2–24.1)	0.91

Data expressed as mean ± standard deviation (95% confidence interval). VAS: visual analog scale.
